# Changes in the intrinsic severity of severe acute respiratory syndrome coronavirus 2 according to the emerging variant: a nationwide study from February 2020 to June 2022, including comparison with vaccinated populations

**DOI:** 10.1186/s12879-023-08869-7

**Published:** 2024-01-02

**Authors:** Boyeong Ryu, Eunjeong Shin, Dong Hwi Kim, HyunJu Lee, So Young Choi, Seong-Sun Kim, Il-Hwan Kim, Eun-Jin Kim, Sangwon Lee, Jaehyun Jeon, Donghyok Kwon, Sungil Cho

**Affiliations:** 1 Epidemiological Investigation and Analysis Task Force, Central Disease Control Headquarters, Korea Disease Control and Prevention Agency (KDCA), 187, Osongsaengmyeong 2-Ro, Osong-Eup, Heungdeok-Gu, Cheongju, Korea; 2 Division of Emerging Infectious Diseases, Bureau of Infectious Diseases Diagnosis Control, Korea Disease Control and Prevention Agency (KDCA), 187, Osongsaengmyeong 2-Ro, Osong-Eup, Heungdeok-Gu, Cheongju, Korea; 3 Department of Infectious Diseases, Clinical Infectious Disease Research Center, National Medical Center, 245, Eulji-ro, Jung-gu, Seoul, Korea; 4 Department of Public Health Science, Graduate School of Public Health, Seoul National University, 1 Gwanak-Ro, Gwanak-Gu, Seoul, 08826 Republic of Korea

**Keywords:** SARS-CoV-2, Intrinsic severity, Case severity rate, Case fatality rate, Variant

## Abstract

**Background:**

As the population acquires immunity through vaccination and natural infection of severe acute respiratory syndrome coronavirus 2 (SARS-CoV-2), understanding the intrinsic severity of coronavirus disease (COVID-19) is becoming challenging. We aimed to evaluate the intrinsic severity regarding circulating variants of SARS-CoV-2 and to compare this between vaccinated and unvaccinated individuals.

**Methods:**

With unvaccinated and initially infected confirmed cases of COVID-19, we estimated the case severity rate (CSR); case fatality rate (CFR); and mortality rate (MR), including severe/critical cases and deaths, stratified by age and compared by vaccination status according to the period regarding the variants of COVID-19 and vaccination. The overall rate was directly standardized with age.

**Results:**

The age-standardized CSRs (aCSRs) of the unvaccinated group were 2.12%, 5.51%, and 0.94% in the pre-delta, delta, and omicron period, respectively, and the age-standardized CFRs (aCFRs) were 0.60%, 2.49%, and 0.63% in each period, respectively. The complete vaccination group had lower severity than the unvaccinated group over the entire period showing under 1% for the aCSR and 0.5% for the aCFR. The age-standardized MR of the unvaccinated group was 448 per million people per month people in the omicron period, which was 11 times higher than that of the vaccinated group. In terms of age groups, the CSR and CFR sharply increased with age from the 60 s and showed lower risk reduction in the 80 s when the period changed to the omicron period.

**Conclusions:**

The intrinsic severity of COVID-19 was the highest in the delta period, with over 5% for the aCSR, whereas the completely vaccinated group maintained below 1%. This implies that when the population is vaccinated, the impact of COVID-19 will be limited, even if a new mutation appears. Moreover, considering the decreasing intrinsic severity, the response to COVID-19 should prioritize older individuals at a higher risk of severe disease.

**Supplementary Information:**

The online version contains supplementary material available at 10.1186/s12879-023-08869-7.

## Background

Coronavirus disease (COVID-19) has various phenotypes of severity ranging from asymptomatic and mild-to-moderate infection to severe and critical condition [1–3]. As the COVID-19 pandemic has continued since 2020 and circulated globally, variants of severe acute respiratory syndrome coronavirus 2 (SARS-CoV-2) have been continuously reported since December 2020 [4, 5], showing differences in characteristics including transmissibility, disease severity, and immune escape [6]. Among the variants of concern designated by the World Health Organization (WHO), delta and omicron have been reported in more than 200 countries, and their phenotypic impacts are different from those of previous viruses, affecting the response to COVID-19 [7].

The delta variant was first reported in South Korea in April 2021 and was detected in more than 50% of cases by July 2021, causing a major epidemic in the winter of 2021 [8]. Several studies have reported that the delta variant had higher transmissibility and clinical severity than the previous circulating viruses [9, 10]. During the delta epidemic, the omicron variant was initially reported in December 2021 and was detected in more than 50% of cases in the 6 weeks since the first report [8]. Globally, the transmissibility of the omicron variant has increased and severity has decreased [11–15]. Despite a lower severity than the previous circulating virus, high transmissibility caused the highest number of infections, leading to the highest number of deaths in South Korea [16].

Despite new variants of SARS-CoV-2, acquired immunity from vaccination and natural infection has reduced the severity of COVID-19. Vaccination is very efficient in preventing severe outcomes and it has been reported that the waning of effectiveness over time is lower than that of infection prevention [12, 17, 18]. Previous studies, where booster doses were evaluated, also showed prevention effects on severe or critical outcomes of COVID-19 [19, 20]. In addition, there are reports that previous infections also affect the severity of COVID-19 infections [12]. With features of substantial escape from immune responses elicited by vaccination or prior infections [21, 22], many countries reported increased cases of reinfection during the omicron period [23, 24].

As the population’s immunity has become heterogeneous owing to vaccination and previous infections, it is more challenging to measure the severity of newly emerged variants of SARS-CoV-2. Although there are limitations as the population’s immunity improves, estimating the intrinsic severity is vital in determining the cause of the change in severity and evaluating its effects on the community [25]. In some studies, intrinsic severity was previously measured among unvaccinated confirmed cases of COVID-19, decreasing the severity of the omicron compared to delta [14, 15]. However, to establish an appropriate and targeted COVID-19 policy consistent with changes in intrinsic severity, a detailed assessment is needed on the difference in age groups, comparison with those vaccinated, and how it affected the community.

South Korea has monitored all confirmed cases using the same indicator since the start of COVID-19 and has measured their severity to identify consistent, representative, and timely COVID-19-specific severity. Therefore, we aimed to evaluate the intrinsic severity trend of COVID-19 among unvaccinated and initial infection cases, according to the variant circulating period, from the early stage of COVID-19. Additionally, we compared the impact of changes in intrinsic severity by age groups and with vaccination status based on the period.

## Methods

### Data sources

We conducted a prospective cohort analysis of the severe, critical cases and deaths-related COVID-19 monitoring in South Korea. Data were collected from the COVID-19 surveillance system and the National Immunization Registry of the Korea Disease Control and Prevention Agency (KDCA). The study included confirmed cases of COVID-19 from February 1, 2020, to June 30, 2022, and monitored their clinical progress until July 23, 2022. Suspected and confirmed cases of reinfection were excluded to calculate the intrinsic severity of non-immunized patients with COVID-19.

### Case definition

A confirmed case was defined as a positive polymerase chain reaction (PCR) test, irrespective of symptoms, or rapid antigen test (from March 14, 2022) with symptoms. All confirmed cases were reported through the KDCA surveillance system according to the Infectious Disease Control and Prevention Act [26]. A severe/critical case was a patient who was treated with high flow oxygenation, non-invasive or invasive ventilation, extracorporeal membrane oxygenation, or continuous renal replacement treatment during hospitalization with COVID-19 isolation, based on the ordinal scale for clinical improvement by the WHO [27]. KDCA monitored all reported severe cases of COVID-19 through its dedicated case management online system and the healthcare resources management system, managed by Ministry of Health and Welfare. Community healthcare centers that managed cases of isolation and hospitals designated for hospitalization of patients with COVID-19 inputted the clinical status of cases in each system. The follow-up of clinical outcomes was implemented during isolation.

Death cases were defined as death reports related to COVID-19, according to the Infectious Disease Control and Prevention Act [26]. The death outcomes were followed up through data reported by the clinician’s statement, regardless of the time point. Deaths may have occurred among those patients who did not meet the criteria for a severe/critical case; that is, if the patients died suddenly before hospitalization or in the long-term care facilities where a high volume of oxygen therapy was not available. Among COVID-19 deaths in Korea, sudden deaths accounted for 24.0% and the proportion of those aged 60 years or above was over 90% [28, 29].

Reinfection was defined as a positive test after 90 days from the initial confirmation of COVID-19, regardless of symptoms, or between 45 and 90 days after the initial confirmation with one of the relevant criteria, such as symptomatic exposure to confirmed patients or overseas travel history.

Vaccination was classified according to the last vaccination status 14 days before the COVID-19 confirmation date. A vaccinated case with one dose of Jcovden (COVID-19 Vaccine from Janssen; Johnson & Johnson Innovative Medicine, Beerse, Belgium) was regarded as complete vaccination.

The period was determined regarding national vaccination initiation and the variants of SARS-CoV-2 circulating. We categorized the period from 14 days after the initiation of nationwide vaccination on February 26, 2021, into pre-vaccination and vaccination before the delta-dominant period ('delta period' hearafter). SARS-CoV-2 variants were identified through genomic surveillance performed by the KDCA [30]. According to the confirmed date of the sample, the delta period began on July 11, 2021, which was the first day of the week that the delta variant was detected in more than 50% of test samples. The omicron dominant period ('omicron period' hearafter) began on January 9, 2022, which was the first day of the week that the omicron variant was detected in more than 50% of test samples in the same week. To ensure the continuity of severity assessment throughout the pandemic, we defined the epidemic period as the duration when a variant was detected in over 50% of test samples. To assess differences in estimations due to overlapping circulating variants, we further analyzed the severity of the weeks in which the variant was detected in over 90% of samples, minimizing the impact of overlapping periods. The delta period was from August 8, 2021 to December 25, 2021, and the omicron period was from January 23, 2022 to end of the study period.

### Statistical analysis

The direct age-standardized case severity rate (aCSR) and age-standardized case fatality rate (aCFR) were calculated by vaccination status and month, with 95% confidence intervals (CIs) based on the gamma distribution. The standard population was the total number of confirmed cases by age group from 0 to 79 years, divided by 10-year intervals, and another group aged ≥ 80 years and over during the entire study period. The severity was evaluated using two distinct indicators: case severity rate (CSR) including severe/critical cases and deaths, and case fatality rate (CFR) including only deaths. As some patients died without meeting treatment criteria for severe/critical cases, severity may be underestimated by including only severe/critical cases. Therefore, the CSR was calculated as the number of severe/critical cases and deaths divided by the number of confirmed cases, and the CFR was calculated only with deaths as the numerator. The CSR and CFR were presented by vaccination status, period, and months. The monthly age-standardized mortality rate (aMR) was calculated as the monthly average number of deaths during each period divided by the population in December 2021. We obtained the vaccinated population for each period by taking the average of the number of vaccinated people at the start and end dates of the period. The statistical analysis was conducted using Excel 2016 (Microsoft Corp., Redmond, WA, USA) and R (version 4.2.1; R Core team, Vienna, Austria).

## Results

From February 1, 2020, to June 30, 2022, 18,359,208 COVID-19 cases were confirmed, and 72,473 cases of reinfection were excluded. Among the 18,286,735 cases, 23,258 severe/critical cases and 24,611 deaths were reported in the COVID-19 monitoring system in South Korea (Additional file 1). Among the 24,611 deaths, only 6,755 (27.5%) were included as severe/critical cases, and other deaths were not included as they were sudden deaths and deaths in places including long-term care facilities where treatment of severe/critical care we defined is not available; described in the methods section.

Table 1 shows the basic characteristics of COVID-19 cases by period. Most of the cases and deaths occurred during the omicron period (96.4% and 72.5% respectively). The mean age of confirmed cases decreased in the omicron period compared to the delta period (37.1 vs. 40.2 years). In contrast, the proportions of older people aged ≥ 80 years among severe/critical cases and deaths increased in the omicron period compared to the delta period (18.6% to 38.5% for severe/critical cases;47.6% to 62.5% for deaths). The vaccination rates increased substantially with the booster from 1.3% in the delta period to 48.3% in the omicron period.

**Table 1:** Characteristics of COVID-19 confirmed cases by period regarding vaccination and circulating variants of SARS-CoV-2

**Class**	**Total**		**Pre-vaccination**		**After-vaccination**					
					**Pre- delta dominant**		**Delta dominant**		**Omicron dominant**	
**Total**	18,286,735	(100.0)	94,179	(100.0)	72,518	(100.0)	493,557	(100.0)	17,626,481	(100.0)
**Sex**										
Male	8,597,627	(47.0)	46,617	(49.5)	37,622	(51.9)	258,770	(52.4)	8,254,618	(46.8)
Female	9,689,108	(53.0)	47,562	(50.5)	34,896	(48.1)	234,787	(47.6)	9,371,863	(53.2)
**Age (years)**										
Mean ± SD	37.3 ± 21.7		45.7 ± 20.6		41.5 ± 19.2		40.2 ± 21.7		37.1 ± 21.7	
0–9	2,184,409	(11.9)	3,894	(4.1)	3,712	(5.1)	45,512	(9.2)	2,131,291	(12.1)
10–19	2,390,072	(13.1)	6,398	(6.8)	5,941	(8.2)	54,157	(11.0)	2,323,576	(13.2)
20–29	2,667,693	(14.6)	14,191	(15.1)	12,362	(17.0)	71,844	(14.6)	2,569,296	(14.6)
30–39	2,691,201	(14.7)	12,453	(13.2)	11,297	(15.6)	72,573	(14.7)	2,594,878	(14.7)
40–49	2,823,960	(15.4)	13,572	(14.4)	12,467	(17.2)	70,814	(14.3)	2,727,107	(15.5)
50–59	2,261,051	(12.4)	17,431	(18.5)	12,930	(17.8)	65,012	(13.2)	2,165,678	(12.3)
60–69	1,855,102	(10.1)	14,642	(15.5)	8,979	(12.4)	69,436	(14.1)	1,762,045	(10.0)
70–79	879,645	(4.8)	7,084	(7.5)	3,380	(4.7)	29,042	(5.9)	840,139	(4.8)
≥ 80	533,602	(2.9)	4,514	(4.8)	1,450	(2.0)	15,167	(3.1)	512,471	(2.9)
**Vaccination**										
None	4,124,629	(22.6)	94,178	(100.0)	70,160	(96.7)	246,166	(49.9)	3,714,125	(21.1)
Partial	209,788	(1.1)	1	(0.0)^*^	1,990	(2.7)	39,389	(8.0)	168,408	(1.0)
Complete	5,441,223	(29.8)	0	(0.0)	368	(0.5)	201,809	(40.9)	5,239,046	(29.7)
Booster	8,511,095	(46.5)	0	(0.0)	0	(0.0)	6,193	(1.3)	8,504,902	(48.3)
**Outcome**										
**Severe/critical**^**a**^^)^	23,258	(0.1)	2,774	(2.9)	1447	(2.0)	8525	(1.7)	10,512	(0.1)
Average per day	26.4		6.9		12.0		46.8		60.4	
Mean age (years± SD)	69.3 ± 15.5		71.1 ± 12.2		65.1 ± 14.1		65.4 ± 15.4		72.6 ± 15.7	
≥ 60	18,170	(78.1)	2,354	(84.9)	987	(68.2)	5,898	(69.2)	8,931	(85.0)
≥ 80	6,560	(28.2)	703	(25.3)	225	(15.5)	1,583	(18.6)	4,049	(38.5)
**Death**	24,611	(0.1)	1,749	(1.9)	348	(0.5)	4,672	(0.9)	17,842	(0.1)
Average per day	27.9		4.3		2.9		25.7		102.5	
Mean age (years± SD)	79.6 ± 12.1		79.3 ± 10.6		76.7 ± 12.1		77.0 ± 12.3		80.3 ± 12.1	
≥ 60	23,063	(93.7)	1,665	(95.2)	318	(91.4)	4,303	(92.1)	16,777	(94.0)
≥ 80	14,492	(58.9)	964	(55.1)	159	(45.7)	2,226	(47.6)	11,143	(62.5)

*COVID-19* coronavirus disease, *SARS-CoV-2 *Severe acute respiratory syndrome coronavirus 2, *SD *Standard deviation

^*^< 0.1

^a^Severe/critical case: Patients who were treated with non-invasive ventilation, high flow oxygenation, invasive ventilation, extracorporeal membrane oxygenation, or continuous renal replacement treatment during isolation from COVID-19

### Intrinsic severity by period

Over the entire period, the aCSR and aCFR were highest at 5.51% (95% CI 5.37–5.65) and 2.49% (95% CI 2.40–2.59) in the unvaccinated group during the delta period (Table 2). Before the delta variant emerged, the intrinsic severity of COVID-19 decreased from 2.49% (95% CI 2.41–2.57) to 2.12% (95% CI 2.01–2.24) for the aCSR and from 1.17% (95% CI 0.12–1.23) to 0.60% (95% CI 0.54–0.67) for the aCFR from the pre- to post-vaccination period. After the delta period, the severity decreased to 0.94 (95% CI 0.92–0.97) for the aCSR and 0.63% (95% CI 0.61–0.64) for aCFR during the omicron period. Although the intrinsic severity of the delta period was the highest, the overall aCSR decreased from 2.06% to 1.93%, and the aCFR increased to a low level from 0.56% to 0.83% (Additional file 2). When assessing epidemic periods with over 90% variant detection, both the delta and omicron periods showed no significant differences in intrinsic severity. In the delta period, the aCSR was 5.50 (95% CI 5.34, 5.66) and the aCFR was 2.55 (95% CI 2.44, 2.66). In the omicron circulating period, the aCSR was 0.92 (95% CI 0.90, 0.94) and the aCFR was 0.61 (95% CI 0.60, 0.63) (Additional file 3).

**Table 2:** Case severity rate^a^^)^ and case fatality rate^b^^)^ by age group, period, and vaccination status (unit: %)

Age group	**Pre-vaccination**		**After-vaccination**						**Delta dominant vs Omicron dominant**											
			**Pre-delta dominant**		**Delta dominant**		**Omicron dominant**													
	**Unvaccinated**	**Vaccinated**	**Unvaccinated**	**Vaccinated**	**Unvaccinated**	**Vaccinated**	**Unvaccinated**	**Vaccinated**	**Unvaccinated**						**Vaccinated**					
									**Risk difference**	**Lower CI**	**Upper CI**	**Risk ratio**	**Lower CI**	**Upper CI**	**Risk difference**	**Lower CI**	**Upper CI**	**Risk ratio**	**Lower CI**	**Upper CI**
**Case Severity Rate (%)**																				
**Total (Crude)**	3.82	-	2.06	3.56	2.66	1.75	0.26	0.11	-2.40	-2.46	-2.34	0.10	0.10	0.10	-1.64	-1.69	-1.59	0.06	0.06	0.07
**Age stand-ardized**	2.49	-	2.12	1.60	5.51	0.93	0.94	0.10	-4.57	-4.71	-4.42	0.17	0.14	0.21	-0.83	-0.86	-0.80	0.10	0.07	0.14
	(2.41—2.57)		(2.01—2.24)	(1.25—2.02)	(5.37—5.65)	(0.90—0.96)	(0.92—0.97)	(0.09—0.10)												
**0–9**	0.00	-	0.00	-	0.02	0.00	0.00*	0.00	-0.01	-0.03	-0.00*	0.24	0.12	0.50	-	-	-	-	-	-
**10–19**	0.00	-	0.05	0.00	0.04	0.00	0.01	0.00^*^	-0.04	-0.06	-0.02	0.14	0.08	0.23	0.00^*^	0.00^*^	0.00^*^	-	-	-
**20–29**	0.09	-	0.19	0.00	0.30	0.03	0.05	0.00^*^	-0.25	-0.30	-0.20	0.17	0.13	0.23	-0.02	-0.04	0.00*	0.12	0.05	0.25
**30–39**	0.33	-	0.42	0.33	1.09	0.09	0.07	0.00^*^	-1.02	-1.12	-0.91	0.06	0.05	0.08	-0.08	-0.11	-0.05	0.05	0.03	0.07
**40–49**	0.69	-	1.03	0.58	2.22	0.16	0.19	0.01	-2.03	-2.19	-1.86	0.09	0.08	0.10	-0.15	-0.19	-0.11	0.06	0.05	0.08
**50–59**	1.79	-	2.12	1.14	5.36	0.46	0.71	0.04	-4.64	-4.94	-4.34	0.13	0.12	0.15	-0.43	-0.49	-0.36	0.08	0.07	0.10
**60–69**	5.46	-	4.71	4.59	14.68	1.95	2.03	0.12	-12.64	-13.37	-11.92	0.14	0.13	0.15	-1.83	-1.94	-1.72	0.06	0.06	0.07
**70–79**	15.33	-	10.94	8.49	31.42	5.44	6.09	0.45	-25.32	-26.82	-23.83	0.19	0.18	0.21	-4.99	-5.27	-4.71	0.08	0.08	0.09
** ≥ 80**	27.69	-	20.54	8.70	44.28	12.58	10.52	1.81	-33.76	-35.45	-32.07	0.24	0.23	0.25	-10.77	-11.37	-10.17	0.14	0.14	0.15
**Case Fatality Rate (%)**																				
**Total (Crude)**	1.86	-	0.47	0.68	1.06	0.84	0.19	0.08	-0.87	-0.91	-0.83	0.18	0.17	0.18	-0.76	-0.79	-0.72	0.09	0.09	0.10
**Age stand-ardized**	1.17	-	0.60	0.31	2.49	0.46	0.63	0.07	-1.87	-1.97	-1.77	0.25	0.20	0.30	-0.39	-0.41	-0.37	0.15	0.10	0.20
	(1.12—1.23)		(0.54—0.67)	(0.17—0.53)	(2.40—2.59)	(0.44—0.48)	(0.61—0.64)	(0.07—0.07)												
**0–9**	0.00	-	0.00	-	0.01	0.00	0.00^*^	0.00	-0.01	-0.01	0.00^*^	0.15	0.04	0.50	-	-	-	-	-	-
**10–19**	0.00	-	0.00	0.00	0.00	0.00	0.00^*^	0.00	0.00^**^	0.00^**^	0.00^*^	-	-	-	-	-	-	-	-	-
**20–29**	0.01	-	0.02	0.00	0.02	0.00^*^	0.02	0.00^*^	-0.00^*^	-0.02	0.01	0.93	0.43	1.99	-0.00*	-0.01	0.00*	0.24	0.03	1.78
**30–39**	0.06	-	0.01	0.33	0.08	0.01	0.02	0.00^*^	-0.06	-0.09	-0.03	0.22	0.14	0.36	-0.01	-0.02	0.00*	0.14	0.04	0.45
**40–49**	0.10	-	0.02	0.00	0.20	0.04	0.09	0.01	-0.12	-0.17	-0.06	0.42	0.31	0.58	-0.03	-0.05	-0.01	0.13	0.07	0.22
**50–59**	0.35	-	0.19	0.00	0.87	0.12	0.36	0.02	-0.51	-0.64	-0.38	0.41	0.34	0.50	-0.10	-0.13	-0.07	0.17	0.12	0.22
**60–69**	1.39	-	0.69	0.54	4.97	0.57	1.08	0.07	-3.89	-4.34	-3.45	0.22	0.19	0.24	-0.50	-0.56	-0.44	0.12	0.10	0.13
**70–79**	7.02	-	3.01	1.89	17.07	2.45	4.13	0.30	-12.94	-14.15	-11.73	0.24	0.22	0.26	-2.15	-2.34	-1.96	0.12	0.11	0.13
** ≥ 80**	21.36	-	12.16	2.72	34.72	8.84	8.75	1.47	-25.97	-27.59	-24.36	0.25	0.24	0.27	-7.37	-7.89	-6.86	0.17	0.16	0.18

*CI* confidence intervals

^*^< 0.01, ^**^< 0.001

^a^Case severity rate: (number of severe/critical cases and deaths among confirmed cases in a specific period)/number of confirmed cases in a specific period * 100 (standard population: total number of confirmed cases in the whole period)

^b^Case fatality rate: (number of deaths among confirmed cases in a specific period)/number of confirmed cases in a specific period * 100 (standard population: total number of confirmed cases in the whole period)

**Fig. 1:**
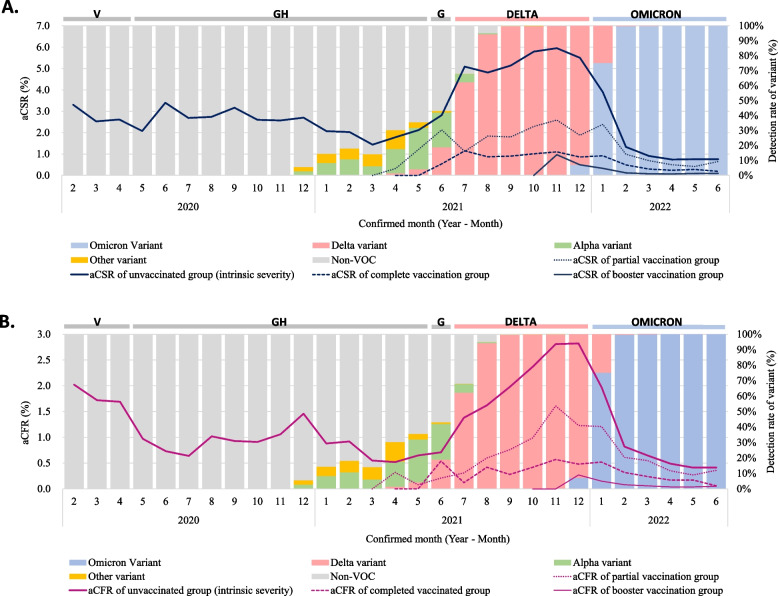
Monthly age-standardized case severity rate (**A**) and case fatality rate (**B**) with the proportion of SARS-CoV-2 variant. aCSR: age-standardized case severity rate; aCFR: age-standardized case fatality rate. **A** Case severity rate: (number of severe/critical cases and deaths among confirmed cases in a specific period)/number of confirmed cases in a specific period *100 (standard population: total number of confirmed cases in the whole period). **B** Case fatality rate: (number of deaths among confirmed cases in a specific period)/number of confirmed cases in a specific period *100 (standard population: total number of confirmed cases in the whole period)

The monthly aCSR and aCFR changed as the variant circulated, as presented in Fig. 1 and Additional file 4. As delta variants were initially reported in April 2021 in South Korea, the aCSR and aCFR of unvaccinated individuals began to increase. However, when omicron variants were first reported in December 2021 in South Korea, the aCSR began to decrease, and the aCFR decreased a month later in the unvaccinated group. There was a clear risk difference between the vaccinated and unvaccinated groups, with the highest risk difference being an aCSR of 3.62% and aCFR of 1.59% in December (aCSR: 5.50% for unvaccinated and 1.88% for partially vaccinated; aCFR: 2.82% for unvaccinated and 1.23% for partially vaccinated).

### Comparison of intrinsic severity with risk of the vaccinated group according to the period

**Fig. 2:**
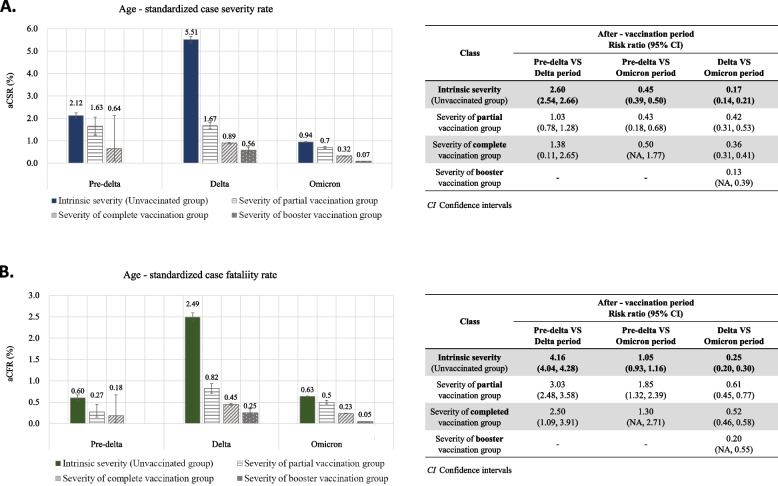
Age-standardized case severity rate (**A**) and case fatality rate (**B**) by period after vaccination. aCSR: age-standardized case severity rate; aCFR: age-standardized case fatality rate. **A** Case severity rate: (number of severe/critical cases and deaths among confirmed cases in a specific period)/number of confirmed cases in a specific period *100 (standard population: total number of confirmed cases in the whole period). **B** Case fatality rate: (number of deaths among confirmed cases in a specific period)/number of confirmed cases in a specific period *100 (standard population: total number of confirmed cases in the whole period)

**Fig. 3:**
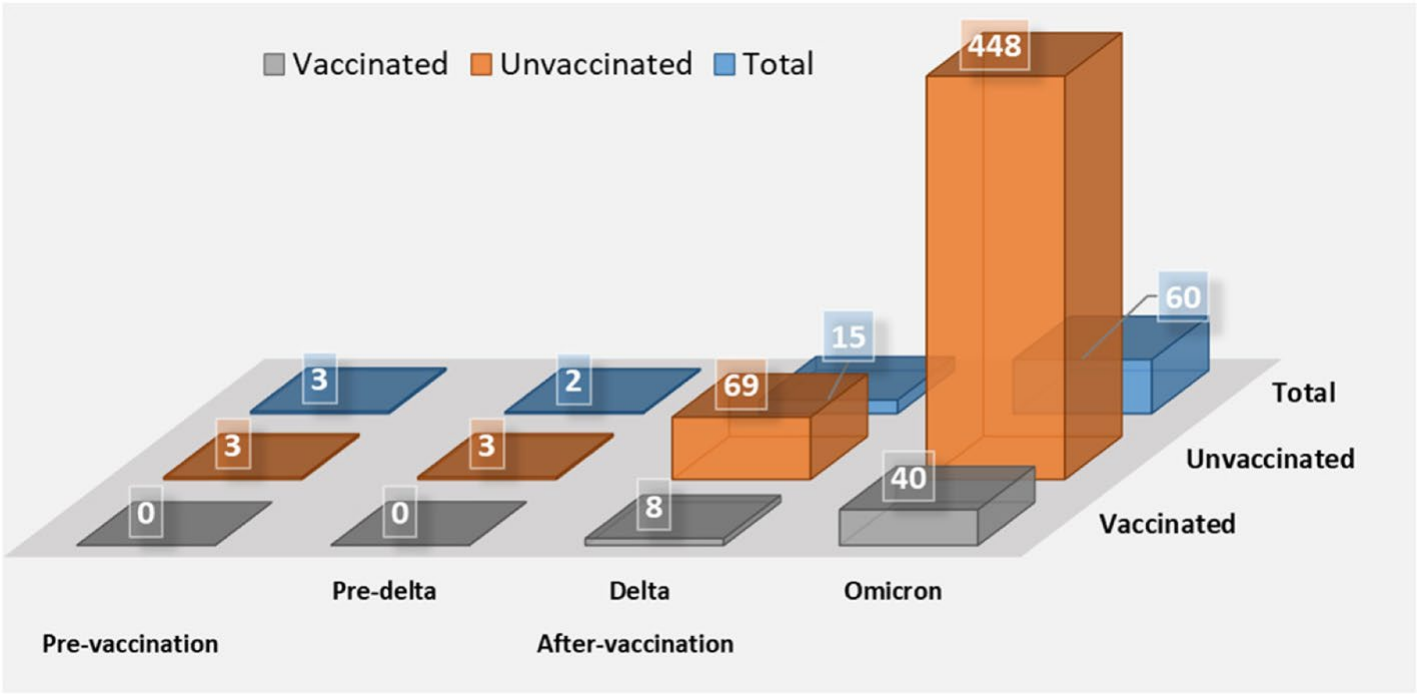
Monthly age-standardized mortality rate per 1,000,000 by period regarding vaccination and circulating variants of SARS-CoV-2. Monthly age-standardized mortality rate: (number of deaths among confirmed cases in a specific period/population of December 2021 *1,000,000)/months (standard population: whole population)

When comparing the unvaccinated group to the vaccinated group, disparities of risk by vaccination status and risk reduction during the omicron and delta periods were observed (Fig. 2, Additional file 2). From the pre-delta to the delta period after vaccination, the aCSR increased from 0.64% (95% CI 0.10–2.12) to 0.89% (95% CI 0.86–0.92) in the completed vaccination group with a lower degree of change than in the unvaccinated group. Similarly, from the delta to the omicron period, the aCSR decreased to 0.32% (95% CI 0.31–0.33) in the vaccinated group with a lower risk ratio than in the unvaccinated group. Only the booster group showed a higher risk ratio of 0.13 times for the aCSR and 0.20 times for the aCFR than in the unvaccinated group from the delta to the omicron period. The risk of the omicron period in the unvaccinated group (aCSR 0.94%, 95% CI 0.92–0.97; aCFR 0.63%, 95% CI 0.61–0.64) was higher than that of the delta period in the completed vaccination group (aCSR 0.89%, 95% CI 0.86–0.92; aCFR 0.45%, 95% CI 0.43–0.47). At the population level, although the intrinsic severity rapidly decreased during the omicron period, the drastic increase in COVID-19 cases related to the high transmissibility of the omicron variant resulted in a six times higher aMR (448) per million people per month than the delta variant (69), and 11 times higher in the unvaccinated group than in the vaccinated group (40) during the same period (Fig. 3).

### Intrinsic severity of COVID-19 by age group and vaccination status

The CSR and CFR by age group and vaccination status during the four periods are summarized in Table 2. The risk of COVID-19, including both the aCSR and aCFR, was the highest during the delta period and the lowest during the omicron period, regardless of vaccination status and age group, except for the 10–19 years and unvaccinated groups. During the delta period, the CSR exceeded 10% in the age group of ≥ 60 years, and the CFR was higher than 10% in the age group of > 70 years. The risk difference of the CSR in the unvaccinated group between the delta and omicron period was the highest at -33.76% (95% CI -35.45 to -32.07) in those aged > 80 years, and the risk ratio was highest at 0.06 (95% CI 0.05–0.08) for those aged in their 30 s. For the CFR of the unvaccinated group, the risk difference was the highest at -25.97% (95% CI -27.59 to -24.36) for those aged > 80 years, and the risk ratio was the highest at 0.22 times (95% CI 0.19–0.24) for those aged in their 60 s and 0.22 (95% CI 0.14–0.36 for those in their 30 s. In the vaccinated group, a similar trend of severity change as in the unvaccinated group was observed. However, the amount of change was low in terms of the risk difference and high in terms of the risk ratio in all age groups.

## Discussion

We assessed how the intrinsic severity of COVID-19 has changed from its emergence to the present, regarding circulating variants of SARS-CoV-2 using nationwide data from South Korea. The results indicated that the intrinsic severity of COVID-19 in the delta period was the highest over the entire period. In contrast, the omicron period had the lowest intrinsic severity and had an 83% lower risk of severe/critical cases with deaths and 75% lower risk of death than that of the delta period. The results of this study are consistent with those found in previous studies in several countries, including South Africa and England [12–15]. However, the decrease was slightly larger than that of a study in England, which demonstrated a 69% decrease [14]. Despite the rapid decrease in the severity of the omicron variant, the severity of the unvaccinated group during the omicron period was slightly higher than that of the fully vaccinated group during the delta period. These results enabled us to compare the intrinsic severity of COVID-19 over the entire period with the overall confirmed cases through consistent measurements of the nationwide cases of COVID-19 in South Korea.

After the vaccination of COVID-19 began, the vaccination group showed less risk fluctuation when the circulating variant changed compared to the unvaccinated group. Although the aCSR in the unvaccinated group increased by 2.6 times when the delta variant emerged and decreased by 1/6 when the omicron variant emerged, the aCSR in the completely vaccinated group increased by 1.4 times and showed a 1/3 decrease in the aCSR, showing a similar trend to the aCFR. A study in Southern California stated that the risk of mechanical ventilation and death related to COVID-19 in the unvaccinated group was significantly reduced during the omicron period than during the delta period and the vaccinated group showed a significant decrease only in deaths, not mechanical ventilation [31]. However, in our study, the vaccinated groups had higher risk reduction in all severe outcomes including severe/critical cases and deaths. This result implies that if the population sustains high levels of immunity from vaccination, even if new variants occur, the community will be less affected by COVID-19. In fact, the intrinsic aCSR and aCFR sharply increased during the pre-delta and delta periods, but the overall aCSR decreased and the aCFR increased to a low level.

In terms of age, intrinsic severity increased with increasing age, especially from the age of 60 years, showing a sharp rise. In previous studies, age has been demonstrated to be the strongest risk factor for severe outcome of COVID-19 [32, 33]. In this study, as the intrinsic severity decreased and the vaccination rate increased, the impact of COVID-19 was concentrated in older people. When the omicron variant emerged, the absolute risk of severe/critical cases and death decreased the most in older people (the high-risk group for COVID-19 in previous studies) [31, 34]. However, the risk ratio of CSR between the omicron and delta periods was the lowest in individuals aged in their 80 s and the highest in those in their 30 s, followed by those in their 40 s. Meanwhile, the risk ratio of CFR, with the risk only for death, did not show a minimum decrease in individuals aged ≥ 80 years, showing a minimum decrease only in those aged in their 40–50 s. The vaccination group was observed to have the lowest risk reduction in those aged ≥ 80 years in both the CSR and CFR among adults aged > 20 years. This result was consistent with that of a study conducted in England, which showed that the magnitude of risk reduction for death during the omicron period compared with the delta period was lower for individuals aged ≥ 80 years than that of other age groups [11]. This disproportionate change was also observed among the ≥ 80 years group, with the proportion of severe/critical cases increasing from 18.6% to 38.5%, despite a decrease in confirmed cases from 3.1% to 2.9% from the delta period to the omicron period. These results imply that as the intrinsic severity of COVID-19 decreases, the response to COVID-19 should focus on the older population with a high risk of severe disease.

Vaccine acceptance may vary by age groups and COVID-19 periods. In one early study, younger generations were more likely to receive vaccination against COVID-19 [35]. In a more recent multinational survey, booster vaccine hesitancy was associated with younger age [36]. However, even in those aged ≥ 60 years, a substantial proportion (24.6%) was less likely to get vaccinated considering the reduced disease severity [36]. COVID-19 vaccination is a critical tool to protect high-risk groups from severe COVID-19 outcomes [37, 38]. While the response against COVID-19 continues as it is an endemic disease, vaccination campaigns for the older age groups should be maintained based on their vulnerability to COVID-19.

Regarding the population, despite a significant decrease in severity during the omicron period, the monthly aMR increased by more than six-fold from 69/million to 448/million in the unvaccinated group due to the high transmissibility of the omicron variant, resulting in an exceedingly high number of confirmed cases. The monthly aMR of the vaccinated group was 8/million and 40/million in the delta and omicron periods, respectively, showing an absolute difference of 61/million and 408/million compared to the unvaccinated group, respectively. With a higher proportion of deaths among those aged ≥ 80 years in the omicron period, this result suggests that even if intrinsic severity is reduced, as the number of confirmed cases increases, the disease burden from COVID-19 could increase particularly among the older people in the community with a low vaccination rate.

As vaccination rates in the country increase and considering the omicron variant’s capacity of high immune escape [17, 18, 39], risk measurements in the overall population tend to represent the immunized population by vaccination and previous infections. Therefore, it is difficult to estimate the intrinsic severity as the outbreak continues [26]. In our study, overall the aCSR and aCFR were compared between those with complete vaccination and the booster group. In South Korea, more than 90% of patients aged ≥ 60 years are vaccinated with one booster; thus, the overall risk reflects that of the booster-vaccinated group [40]. Among those who tested positive for COVID-19, the clinical severity and mortality varied by multiple factors including the vaccination history and patients’ age and health status [19, 32, 33]. Therefore, the intrinsic severity of virus variants should be monitored differentially in the unvaccinated group by patients’ characteristics.

Since our study included overall confirmed cases in South Korea and monitored the clinical status with the same indicators over the entire period, the severity of COVID-19 could be compared consistently. Additionally, extensive tests through the rapid scaling of diagnostic capacity from central laboratories to regional laboratories; free-of-charge tests; fast and high accessibility of testing, including drive-through and walk-through screening centers; large and periodic screening tests in high-risk groups; and proactive contact tracing enabled the enhanced understanding of infection severity and fatality rates [41].

However, our study had some limitations. First, confirmation tests, including the PCR and rapid antibody test, could have false positive and false negative results, especially among those with underlying medical conditions. A comprehensive review of the potential sources of errors has been described in previous studies [42, 43]. Second, the number of cases was not adjusted to the COVID-19 test coverage. According to a sero-epidemiological study in Korea, the estimated prevalence of previous SARS-CoV-2 infection was 57.65% compared to the reported cumulative incidence of 38.15%, implying that approximately a third of infected cases were not identified, and thus that our estimates of the CSR and CFR may have been overestimated [44]. Specifically by the age group, the proportion of unreported infections was highest in the 50 s age group (47.9%) followed by the 60 s (45.0%) and 70 s (40.5%), and lowest in the 80 s (12.9%). This findings suggests that the overestimation of observed severity of our study in the 50 s, 60 s, and 70 s age groups was more pronounced, while the 80 s showed a comparatively lower degree of overestimation. Consequently, these results imply that disparities in severity between 80 s and other age groups could be more extensive than observed in our study. Third, we may have included unidentified reinfections in the analysis. Assessing the exact number of reinfections is difficult and requires a different study design, such as repeated follow-up testing regardless of symptoms. According to the studies of reinfection in Korea, most reinfection cases were reported in the omicron period, showing under 0.5% until week 14, 2022 and 0.5%–3.0% until week 26, 2022 of confirmed cases [45, 46]. Although the prevention of severe outcomes by past infection showed a varing spectrum, more reports indicated that reinfection prevented severe outcomes or showed similar severity with primary infection [45–54]. Therefore, in our study, we assumed a higher possibility of including unknown reinfection cases during the omicron period, and as a result, the CSR and CFR in this period could be underestimated or remain the same. Lastly, the time from vaccination to confirmed COVID-19 was not considered; therefore, the waning efficacy of vaccination may have affected the overestimation of the risk of vaccinated groups.

## Conclusion

Although the intrinsic severity of COVID-19 fluctuated with the emergence of new variants, vaccination can mitigate the severity of COVID-19. Additionally, as the severity decreased according to the characteristics of the circulating virus and vaccination status, response to COVID-19 should focus on older adults, who are at a high-risk of severe disease. With the increasing diversity of the immunized status of the population, the intrinsic severity of our study can be considered a baseline risk for comparison with changing severity, which may be vital for determining attributable factors when monitoring changes in severity.

## Supplementary Information

**Additional file 1. **The number of confirmed cases, including severe/critical cases and deaths.

**Additional file 2. **Age-standardized case severity rates and case fatality rates by period regarding vaccination and circulating variant of SARS-CoV-2 (%).

**Additional file 3. **Age-standardized case severity rates and case fatality rates during weeks with SARS-CoV-2 variants detected in over 90% of test samples (%).

**Additional file 4. **Monthly age-standardized case severity rates and case fatality rates.

## Data Availability

The individual-level data are not available to be shared publicly. However, descriptive statistics of coronavirus disease (COVID-19) are available online (Open data, Korea Disease Control and Prevention Agency; https://ncov.kdca.go.kr/).

## Abbreviations

COVID-19: Coronavirus disease
CSR: Case severity rate
CFR: Case fatality rate
MR: Mortality rate
aCSR: Age-standardized case severity rate
aCFR: Age-standardized case fatality rate
a MR: Age-standardized mortality rate
SARS-CoV-2: Severe acute respiratory syndrome coronavirus 2
WHO: World Health Organization
KDCA: Korea Disease Control and Prevention Agency
CI: Confidence intervals
PCR: Polymerase chain reaction
IRB: Institutional Review Board
