# Supplementary figures and images for: Changes in the intrinsic severity of severe acute respiratory syndrome coronavirus 2 according to the emerging variant: a nationwide study from February 2020 to June 2022, including comparison with vaccinated populations
Source: BMC Infect Dis. 2024 Jan 2;24:1. doi: 10.1186/s12879-023-08869-7 (PMC10759357; doi:10.1186/s12879-023-08869-7)

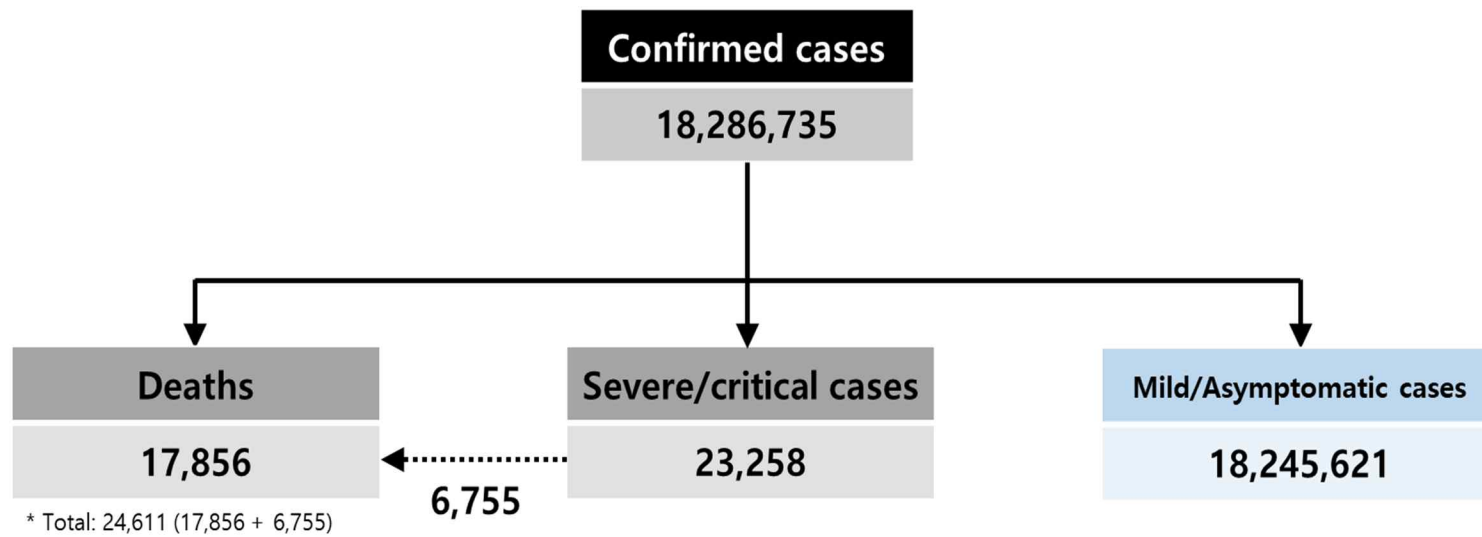

**Additional file 1.** The number of confirmed cases, including severe/critical cases and deaths.

Supplement: Supplementary file 1 — Additional file 1. The number of confirmed cases, including severe/critical cases and deaths. [file 12879_2023_8869_MOESM1_ESM.pdf]
