# Supplementary material for: Changes in the intrinsic severity of severe acute respiratory syndrome coronavirus 2 according to the emerging variant: a nationwide study from February 2020 to June 2022, including comparison with vaccinated populations
Source: BMC Infect Dis. 2024 Jan 2;24:1. doi: 10.1186/s12879-023-08869-7 (PMC10759357; doi:10.1186/s12879-023-08869-7)
Supplement: Supplementary file 2 — Additional file 2. Age-standardized case severity rates and case fatality rates by period regarding vaccination and circulating variant of SARS-CoV-2 (%). [file 12879_2023_8869_MOESM2_ESM.pdf]

Additional file 2. Age-standardized case severity rates and case fatality rates by period regarding vaccination and circulating variant of SARS-CoV-2 (%).

Vaccination status	Total	Pre-vaccination	After-vaccination		
			Pre-delta dominant	Delta dominant	Omicron dominant
**Case severity rate (95% CI)**					
**Total**	0.22 (0.22, 0.23)	2.49 (2.41, 2.57)	2.06 (1.95, 2.17)	1.93 (1.89, 1.97)	0.14 (0.14, 0.15)
Unvaccinated	1.66 (1.64, 1.68)	2.49 (2.41, 2.57)	2.12 (2.01, 2.24)	5.51 (5.37, 5.65)	0.94 (0.92, 0.97)
Partial vaccination	0.94 (0.89, 0.98)	-	1.63 (1.26, 2.06)	1.67 (1.53, 1.83)	0.7 (0.65, 0.75)
Complete vaccination	0.45 (0.44, 0.46)	-	0.64 (0.10, 2.12)	0.89 (0.86, 0.92)	0.32 (0.31, 0.33)
Booster vaccination	0.07 (0.07, 0.07)	-	-	0.56 (0.42, 0.72)	0.07 (0.07, 0.07)
**Case fatality rate (95% CI)**					
**Total**	0.13 (0.13, 0.14)	1.17 (1.12, 1.23)	0.56 (0.50, 0.62)	0.83 (0.81, 0.85)	0.10 (0.10, 0.10)
Unvaccinated	0.82 (0.80, 0.84)	1.17 (1.12, 1.23)	0.60 (0.53, 0.67)	2.49 (2.40, 2.59)	0.63 (0.61, 0.64)
Partial vaccination	0.51 (0.48, 0.55)	-	0.27 (0.15, 0.45)	0.82 (0.71, 0.94)	0.50 (0.46, 0.54)
Complete vaccination	0.29 (0.28, 0.29)	-	0.18 (0.02, 0.67)	0.45 (0.43, 0.47)	0.23 (0.23, 0.24)
Booster vaccination	0.05 (0.05, 0.05)	-	-	0.25 (0.17, 0.34)	0.05 (0.05, 0.05)

SARS-CoV-2 Severe acute respiratory syndrome coronavirus 2, *CI* Confidence Intervals
