# Supplementary material for: Changes in the intrinsic severity of severe acute respiratory syndrome coronavirus 2 according to the emerging variant: a nationwide study from February 2020 to June 2022, including comparison with vaccinated populations
Source: BMC Infect Dis. 2024 Jan 2;24:1. doi: 10.1186/s12879-023-08869-7 (PMC10759357; doi:10.1186/s12879-023-08869-7)
Supplement: Supplementary file 3 — Additional file 3. Age-standardized case severity rates and case fatality rates during weeks with SARS-CoV-2 variants detected in over 90% of test samples (%). [file 12879_2023_8869_MOESM3_ESM.pdf]

Additional file 3. Age-standardized case severity rates and case fatality rates during weeks with SARS-CoV-2 variants detected in over 90% of test samples (%).

Vaccination status	Delta detected over 90%	Omicron detected over 90%
**Case severity rate (95% CI)**		
**Total**	1.84 (1.81, 1.88)	0.14 (0.14, 0.14)
Unvaccinated	5.50 (5.34, 5.66)	0.92 (0.90, 0.94)
Partial vaccination	1.72 (1.56, 1.90)	0.68 (0.63, 0.73)
Complete vaccination	0.88 (0.85, 0.91)	0.31 (0.30, 0.32)
Booster vaccination	0.50 (0.31, 0.75)	0.07 (0.07, 0.07)
**Case fatality rate (95% CI)**		
**Total**	0.82 (0.79, 0.84)	0.10 (0.10, 0.10)
Unvaccinated	2.55 (2.44, 2.66)	0.61 (0.60, 0.63)
Partial vaccination	0.85 (0.72, 0.99)	0.49 (0.45, 0.54)
Complete vaccination	0.45 (0.43, 0.47)	0.23 (0.22, 0.24)
Booster vaccination	0.28 (0.15, 0.45)	0.05 (0.05, 0.05)

SARS-CoV-2 Severe acute respiratory syndrome coronavirus 2, *CI* Confidence Intervals
