# Supplementary material for: Changes in the intrinsic severity of severe acute respiratory syndrome coronavirus 2 according to the emerging variant: a nationwide study from February 2020 to June 2022, including comparison with vaccinated populations
Source: BMC Infect Dis. 2024 Jan 2;24:1. doi: 10.1186/s12879-023-08869-7 (PMC10759357; doi:10.1186/s12879-023-08869-7)
Supplement: Supplementary file 4 — Additional file 4. Monthly age-standardized case severity rates and case fatality rates. [file 12879_2023_8869_MOESM4_ESM.pdf]

Additional file 4. Monthly age-standardized case severity rates and case fatality rates

Year-Month		Age-standardized case severity rate (%)				Age-standardized case fatality rate (%)			
		Unvaccinated	Vaccinated			Unvaccinated	Vaccinated		
			Partial	Complete	Booster		Partial	Complete	Booster
2020	2	3.30				2.02			
	3	2.53				1.72			
	4	2.62				1.69			
	5	2.08				0.97			
	6	3.40				0.73			
	7	2.69				0.64			
	8	2.74				1.02			
	9	3.17				0.93			
	10	2.60				0.91			
	11	2.57				1.06			
	12	2.70				1.46			
2021	1	2.07				0.88			
	2	2.03				0.92			
	3	1.44	0.00			0.55	0.00		
	4	1.79	0.32			0.52	0.32		
	5	2.12	1.22	0.00		0.65	0.08	0.00	
	6	2.82	2.13	0.54		0.71	0.21	0.54	
	7	5.09	1.12	1.15		1.38	0.31	0.12	
	8	4.81	1.84	0.87		1.62	0.60	0.42	
	9	5.14	1.80	0.90		1.98	0.76	0.28	
	10	5.79	2.29	1.01	0.00	2.37	0.99	0.42	
	11	5.95	2.59	1.10	0.97	2.81	1.61	0.57	0.00
	12	5.50	1.88	0.86	0.50	2.82	1.23	0.48	0.27
2022	1	3.89	2.39	0.92	0.33	1.97	1.21	0.52	0.15
	2	1.33	0.99	0.49	0.12	0.82	0.61	0.32	0.08
	3	0.91	0.69	0.30	0.08	0.65	0.55	0.24	0.06
	4	0.74	0.50	0.24	0.07	0.49	0.35	0.17	0.04
	5	0.76	0.42	0.28	0.09	0.41	0.27	0.17	0.04
	6	0.76	0.65	0.19	0.10	0.41	0.36	0.06	0.05
